# The Influence of Abdominal Adiposity and Physical Fitness on Obesity Status of Portuguese Adolescents

**DOI:** 10.3390/ijerph191811213

**Published:** 2022-09-07

**Authors:** Samuel Gonçalves Almeida da Encarnação, Pedro Flores, David Magalhães, Gil Afonso, Albino Pereira, Rui Brito Fonseca, Joana Ribeiro, Sandra Silva-Santos, José Eduardo Teixeira, António Miguel Monteiro, Ricardo Ferraz, Luís Branquinho, Pedro Forte

**Affiliations:** 1 Research Centre in Sports Sciences, Health Sciences and Human Development (CIDESD), 5001-801 Vila Real, Portugal; 2 Higher Institute of Educational Sciences of the Douro (ISCE Douro), 4560-708 Penafiel, Portugal; 3 CI-ISCE/ISCE Douro, 4560-708 Penafiel, Portugal; 4 Agrupamento de Escolas de Vilela, 4580-651 Paredes, Portugal; 5 Escola Superior de Desporto e Lazer, Instituto Politécnico de Viana do Castelo, 4900-347 Viana do Castelo, Portugal; 6 Department of Sport Sciences, Polytechnic Institute of Bragança (IPB), 5300-253 Bragança, Portugal; 7 Department of Sports Sciences, University of Beira Interior (UBI), 6201-001 Covilhã, Portugal

**Keywords:** BMI, overweight/obesity, adolescence, Portugal

## Abstract

The aim of this study was to verify the relationship between abdominal adiposity and physical fitness in the obesity status of Portuguese adolescents. BMI, abdominal adiposity, and physical fitness tests from the FITescola^®^ were evaluated in a total of 654 Portuguese adolescents, aged 10-19 years old—male: *n* = 334 (51%), female: *n* = 320 (49%). For the final model, BMI was positively related with age (*β =* 0.25), abdominal adiposity (*β =* 0.313), horizontal jump (*β =* −0.100), 40 m maximal velocity (*β =* 0.274), and aerobic fitness (*β =* −0.250, *R*^2^ = 0.75, *F* = 382.2, and *p* = 0.0001). We conclude that higher age and abdominal adiposity are positively related with a higher adolescent BMI. Furthermore, lower horizontal jump distances and worse times on the 40 m maximal velocity were inversely related with a higher adolescent BMI, and lower aerobic fitness was inversely related with a higher adolescent BMI.

## 1. Introduction

Obesity is a growing world public health problem, and it is highly prevalent in all phases of life [1]. According to data from the World health Organization (WHO), obesity, accompanied by the use of alcohol and tobacco and low physical exercise practice, is one of the most significant and worsening health-related conditions of adolescent health [2]. In Portugal, the data from the last survey in 2018 show that around 34% of the overall population had obesity, and about 18% of children and 24% of the adolescents had pre-obesity [3]. A high body mass index (BMI) during childhood and adolescence is related to various non-communicable chronic diseases, such as diabetes [4,5] and cardiovascular diseases [6], and also leads to a low quality of life [7], and, if not, these complications are extended into later life [8]. Another component of obesity is abdominal obesity, characterized by a high accumulation of fat mass in the abdominal region, which, in turn, increases the risk of cardiovascular disease [9], cancer [10], and diabetes [11], reduces the immune system function, and increases inflammatory levels [12,13]. These factors will result in a public health problem and evaluations are required to identify the influencing factors and, thus, define strategies for community interventions [2].

Many factors have an effect on body weight control and obesity evolution, such as good nutritional habits [14], avoiding the use of tobacco/alcohol [15], good sleep quality [16], and keeping good physical fitness [17]. Several studies have analyzed the variables that are associated with obesity in adolescents, such as physical fitness in Chinese adolescents [18], exacerbation caused by the use of electronic media by Austrian adolescents [19], and poor sleep quality in Chinese adolescents [16]. In a cross-sectional study, with data from 851 Colombian adolescents, a positive association was found between low physical activity levels and high insulin resistance in the obesity status of adolescents [20]. In addition, the evidence shows that during adolescence adolescents change their behavior significantly, adopting an unhealthy lifestyle, including the use of technology, poor nutritional diet, and low physical activity, which increase the risk of overweight and obesity; shows the importance of monitoring these variables and other health-related variables during adolescence [21].

However, with regard to the first point, the majority of studies concentrate their analyses on physical activity levels and abdominal adiposity, but do not measure directly the contribution of physical fitness levels and abdominal adiposity to the obesity status of adolescents [18,19,22,23]. In the second point, there have been no studies in Portugal analyzing the influences of abdominal obesity and physical fitness on the obesity status of adolescents, and when some studies have investigated European adolescents, the analysis concentrates only on the obesity status and physical activity levels but not on the relationship of the physical fitness levels with the obesity status of the adolescents [18,24,25]. However, regarding the physical fitness evaluations, it is possible to identify in the literature different batteries, such as Eurofit^®^ [26,27], Fitnessgram^®^ [28], and the Alpha-fit test battery [29]. However, in Portugal, schools use the so-called FITescola^®^ [30].

This investigation helps to explore how abdominal adiposity and physical fitness are meaningfully related and, consequently, how they can influence the obesity status of the Portuguese adolescent population. Thus, the objective of this study was to verify the relationship between abdominal adiposity and physical fitness in the obesity status of Portuguese adolescents. In this sense, from evidence from studies with adolescents in other European localities [31,32], we raise the following two hypotheses: (1) abdominal adiposity has a positive relationship with obesity status, and (2) physical fitness has a negative relationship with the obesity status of Portuguese adolescents.

## 2. Materials and Methods

### 2.1. Study Subjects

This is an observational, prospective, and cross-sectional study which aims to evaluate the prevalence of obesity by body mass index (BMI) in adolescents from both genders, and the impacts on the abdominal adiposity and physical fitness in the adolescents’ obesity status (BMI). We used data from FITescola^®^, a Portuguese project which aims to promote healthy behavior in children and adolescents. The data collection included the BMI, abdominal adiposity, and physical fitness tests from the FITescola^®^ that happened in September 2021 on the Paredes council. Thus, in total, 654 adolescents aged between 10–19 years old—male: 334 (51%), female: *n* = 320 (49%)—were selected to participate in this study if it was convenient. In addition, the eligibility criteria considered adolescents of both genders, free of any disabling condition, and with ages ranging between 10-19 years old, adopting the WHO cohort cut-offs for adolescents, according to which the WHO divides adolescence into the following three categories: 1) pre-adolescence (10–14 years old) and 2) adolescence (15–19 years old); thus, this age range was selected respecting the WHO cut-offs [33]. The characteristics of the sample are provided in Table 1 below.

### 2.2. Ethical Aspects

This study was approved by the Scientific Board of the Higher Institute of Educational Sciences of the Douro (PF:10.2021). Prior to the study, the parents (or legal guardians) of all minor participants were asked to sign a written informed consent.

### 2.3. Data Collection

In our study, the following data were collected:

For obesity status, the body mass index (BMI) and abdominal adiposity abdominal circumference were collected [34]. Physical fitness was evaluated throughout with the horizontal jump (HJ), the 40 m maximal velocity test for the maximal speed velocity in 40 m, and the Yo-Yo test. The validity and reliability of the physical fitness tests in the FITescola**^®^** battery had previously been confirmed for physical education and sports [35,36].

### 2.4. BMI

(1) The body weight was collected with the subject barefoot and wearing light clothes, standing up, and waiting for the score of the brand scale to stabilize. A brand scale with a precision of 100 g was used.

(2) The height was collected with the subject barefoot, feet together, and with back touching the stadiometer scale. The stadiometer hod was put at the top of the subject’s head to compress the higher part of the head (vertex). A stadiometer with a precision of millimeters was used.

(3) The BMI was verified through the ratio of body weight/height squared (in kg/m^2^). The cut-offs for the risk of cardiovascular disease were taken following the normative of the WHO for adolescents [34].

### 2.5. Abdominal Adiposity

Abdominal adiposity was verified by measuring body circumference. The subject was asked to stand up with a relaxed belly and to lift the shirt to expose the area to be measured. Then, the measuring tape was placed around the waist, in the horizontal plane, 1 cm above the top of the iliac crests. The subject was asked to perform a normal expiration. The value obtained at the end of expiration was recorded in cm and with a precision tape measure of 0.1 cm. Two attempts were performed and taken, and the average of the two measurements was the final result of the evaluation The cut-offs for the risk of cardiovascular disease were taken following the normative of the Journal American College of Cardiology for adolescents’ abdominal circumference [37].

### 2.6. Horizontal Jump

To evaluate the horizontal jump, a horizontal line was drawn at the starting point and reference lines were drawn every 10 cm (1 m after the starting line). A measuring tape with an accuracy of millimeters was placed perpendicular to the horizontal lines to facilitate the measurement of the distance reached. The subject was positioned standing behind the line that marked the starting point, with feet shoulder-width apart. Starting from the standing position, in a continuous movement, the subject had to bend the knees, pull the arms behind, and jump as far as possible. Distances were measured from the starting point to the heel. Two attempts were performed to take the best result of the two evaluations in cm [38].

### 2.7. 40 m Maximal Velocity

For the evaluation of the running speed of 40 m at maximal velocity, the test of maximum speed in 40 m was applied. A 3 min warm-up for general muscle activation was performed to avoid injuries during the test. Two signaling cones were used to identify the initial and final points of the test. The subject was positioned standing behind the line that marked the starting point, with the lower limbs in the anteroposterior distance and the trunk slightly inclined forward. At the sign of “prepare, now!” from the evaluator, the stopwatch was started with an accuracy of 0.1 s, and the evaluated subject had to start a run at the highest possible speed. When the student crossed the finish line, the timer was stopped. Two trials were given, and the value recorded was the best result of the two trials [39].

### 2.8. Aerobic Fitness

For the assessment of aerobic fitness, the Yo-Yo test was applied. Two cones were used to delimit the space of 20 m between the beginning and the end of the test. The subject was positioned behind the starting line, and at the signal of “prepare, now!”, the subject started the race and had to touch the final line of 20 m when he heard the sound signal generated by the audio. At the sound signal, the subject had to then reverse the running direction and run to the other end. If the subject reached the line before the beep, he had to wait for the new beep to run in the opposite direction. Ideally, the subject should control the running pace to reach the end of the 20 m just before the beep. The audio signal helped the student to mark the speed during the course. Initially, the speed was lower (8.5 km/h) and progressively increased (0.5 km/h every minute; 1 min is equal to one stage) up to a maximum of 120 routes. The sound signal indicated the end of a 20 m course, and a triple sound signal indicated the end of each stage. When the subject could not reach the final line of the course, he had to immediately reverse the direction of his run, even if he had not reached the line. The subject had to remain in the test as long as possible and stop when he could no longer reach the line before the audio signal on two, not necessarily consecutive, occasions. The first foul counted towards the final score. The result was the highest number of laps performed [40].

### 2.9. Statistical Analysis

The statistical procedures were performed using the R version 4.0.0 program within the Rstudio environment. The sample characterization data were described in absolute values and percentages. For the sample characteristic variables, the chi-square test for simple proportions (*X*²) was applied, and the effect size calculation was performed by the Cramer’s V effect size [41]. Then, a multiple regression analysis (R) was conducted to estimate the 95% confidence intervals (CIs) between the explanatory variables of abdominal adiposity and physical fitness and the obesity status of adolescents. Initially, the variables were hierarchically placed in the model, based on the literature investigated by the researcher [42], who chose not to use the stepwise method in the study in question. According to [42], the stepwise method is convenient when the exploratory analysis has data that the researcher is not aware of; otherwise, this method may mathematically exclude variables that have statistical weight from the model, and it is recommended to avoid it if the researcher has good science and mastery of the knowledge. The sample size reached the minimum margin of 40 samples established by [43] to find a large effect size TE (0.80) with statistically significant power [43]. For the effect size of the model, the determination coefficient R^2^ was used, in which the R² is obtained through the root of the R value of the multiple regression [44]. The significance level adopted will be *p* < 0.05 [42].

## 3. Results

Table 2 shows the characteristics of the adolescents in this study. A total of 370 adolescents participated in this study. The sample presented a mean age of 15.7 ± 1.21 years old (y/o). There were differences found in the overweight prevalence for both genders, (overweight male: *n* = 55 (18%) vs. obese male: *n* = 37 (11%), *X*^2^ = 34.66, *df* = 1, large ES, *V* = 0.50, *p* < 0.001; overweight female: *n* = 65 (20%) vs obese female: *n* = 36 (12%), *X*^2^ = 36.67, *df* = 1, large ES, *V* = 0.50, *p* < 0.001). We found no differences between gender for overweight and obesity *p* > 0.05. Therefore, the subgroups of 11, 12, 13, 15, and 18 y/o for both genders presented more adolescents for both genders with overweight (11 y/o: *n* = 20 (25%); 12 y/o: *n* = 11 (21%); 13 y/o: *n* = 22 (26%); 15 y/o: *n* = 24 (21%), *X*^2^ = 46.81, *df* = 1, large *ES*, *V* = 0.57, *p* < 0.001). Additionally, the subgroups of 10, 11, 13, and 14 y/o presented more adolescents for both genders with obesity (10 y/o: *n* = 11 (17%); 11 y/o: *n* = 11 (14%); 13 y/o: *n* = 12 (14%); 14 y/o: *n* = 9 (12%), *X*^2^ = 37.05, *df* = 1, large *ES*, *V* = 0.83, *p* = < 0.001). Therefore, we found differences between groups for the abdominal adiposity, in which females presented a higher percentage of adolescents at risk (females: *n*= 164 (53%) vs males: *n* = 45 (16%), *X*^2^ = 88.311, *df* = 1, large *ES*, *V* = 0.98, *p* = < 0.001), and the age categories of 11 and 15 years old for both genders presented the greater percentage of adolescents with elevated abdominal adiposity, respectively (11 years old: *n* = 43 (7%), and 15 years old: *n* = 64 (10%), *X*^2^ = 86.406, *df* = 1, large *ES*, *V* = 1, *p* = < 0.001).

Table 3 shows the results from the hierarchical multiple linear regression analysis of the abdominal adiposity, physical fitness, and obesity status of adolescents shown in Table 2. In total, for the first model, adjusted for age, the obesity status evaluated by BMI was significantly related with age (95% CI = 0.139 to 0.314, *β* = 0.25, *p* = <0.001), abdominal adiposity (95% CI = 0.305 to 0.337, *β* = 0.313, *p* = < 0.001), horizontal jump (95% CI = 0–0.163 to −0.0008, *β* = − 0.100, *p* = 0.02), 40 m maximal velocity (95% CI = 0.025 to 0.544, *β* = 0.274, *p* = 0.04), and aerobic fitness (95% CI = −0.038 to −0.011, *β* = −0.250, *p* = 0.0001), (*R*^2^ = 0.75, *F* = 382.2, *p* = 0.0001). The second model was adjusted for gender, and an association was not found between gender and the adolescents’ BMIs *p* > 0.05. No differences were found between the sums of square variances of the two models; thus, model 1 was chosen due to the better adjustment to the adolescents’ BMIs.

**Figure 1:**
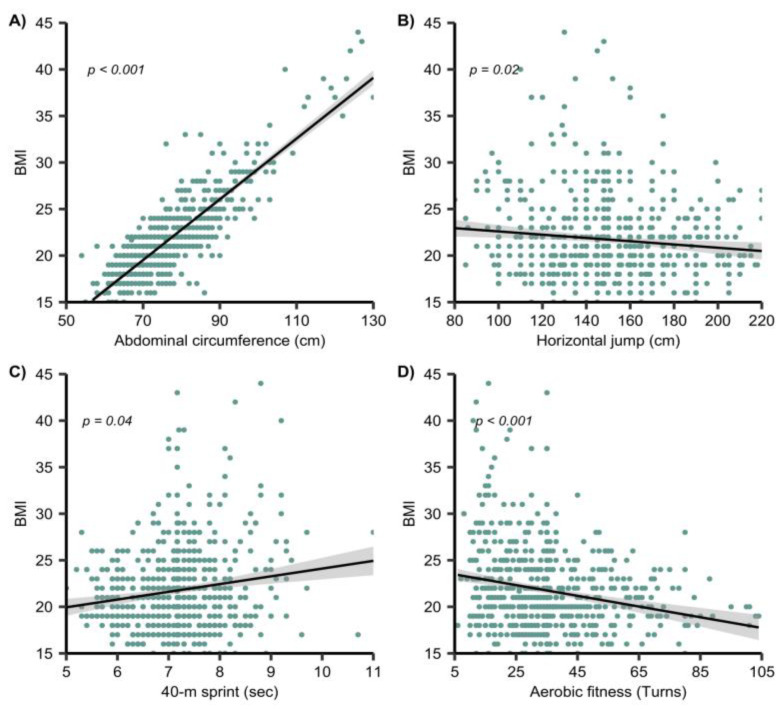
Data visualization of the physical fitness tests in model 1: (**A**) abdominal adiposity; (**B**) horizontal jump; (**C**) 40 m velocity, 40 m maximal velocity; (**D**) aerobic fitness. Statistical significance at *p <* 0.05.

The results from the hierarchical multivariate regression analyses are also described in the data visualization in Figure 1 below.

## 4. Discussion

This study aimed to verify the relationship between abdominal adiposity and physical fitness in the obesity status of Portuguese adolescents. Moreover, in our study the predictor variables organized hierarchically in the model were adequate to explain the adolescents’ BMIs. Moreover, our two hypotheses were confirmed, in which (1) abdominal obesity was significantly positively related to the adolescents’ BMIs and (2) horizontal jump, 40 m maximal velocity, and aerobic fitness were significantly inversely related with the adolescents’ BMIs. The adjustments in model 1 showed that the adolescent’s age was also positively related and contributed 25% to the adolescent’s BMI (95% CI = 0.139 to 0.314, *β =* 0.25). Moreover, similarly to our study, a recent investigation with Italian children and adolescents aged between 5–13 years old found that the oldest category of adolescents (11–13 years old) for both genders presented a significant positive association with obesity [45]. Furthermore, higher ages are considered a stronger isolated factor for obesity prevalence in children [46] and adolescents [47]. Additionally, other factors also contribute to improving the obesity prevalence in adolescents, such as low physical activity levels [47], excessive use of smartphones [48], and inadequate fruit and vegetable intake [49].

In the present study, abdominal obesity was evaluated by the abdominal circumference, which was significantly positively related and contributed to 31% of an adolescent’s BMI (95% CI = 0.305 to 0.337, *β =* 0.313), showing that central obesity contributed to the overall obesity status of adolescents. Furthermore, the gender analysis showed that the female group presents the higher number of subjects with elevated abdominal adiposity (female: *n* = 164 (53%) vs male: *n* = 45 (16%), and for the analysis between both genders, the ages of 11 and 15 years old presented the greater number of adolescents with elevated abdominal obesity (11 y/o: *n* = 43 (7%), 15 y/o: *n* = 64 (10%). Abdominal adiposity is considered a determinant marker of cardiovascular risk/metabolic deregulation in adolescents [9] and is also positively related to low self-esteem and body dissatisfaction in children and adolescents [50], and in some cases, it can occur without the presence of a high BMI but by the specific body regions with high body fat concentrations [6,51].

The lower limb muscle strength and power were analyzed throughout the horizontal jump and 40 m maximal velocity tests, in which the horizontal jump was inversely related and contributed 10% to an adolescent’s BMI (95% CI = 0–0.163 to −0.0008, *β =* −0.100), and the time in seconds in the 40 m maximal velocity test was also significantly inversely related and contributed 27% to an adolescent’s BMI (95% CI = 0.025 to 0.544, *β =* 0.274). Additionally, similarly to our study, an investigation in Kosovo, southeast Europe, with 353 children and adolescents of both genders, showed that a low horizontal jump and a longer time in a 20 m sprint had a significantly increased relative risk, and longer time in the 20 m sprint signified overweight and obesity, respectively (*RR* = 0.99, *p* < 0.001), (*RR* = 1.35, *p* = 0.04) [52]. Lower limb muscle strength and power are positively related to good bone health and are inversely related with cardiovascular disease and metabolic syndrome in children and adolescents [53]. Moreover, keeping satisfactory lower limb power levels during early life and adolescence leads to good physical functionality in daily living activities (DLA), such as walking/running efficiently and increasing daily movements, thus helping to reduce sedentary behavior and decrease the risk of metabolic syndrome as well as improving the quality of life [54].

Moreover, the analysis of the aerobic fitness throughout the Yo-Yo test was inversely related and contributed to 25% of an adolescent’s BMI (95% CI = −0.038 to −0.011, *β =* −0.250). A study enrolling 3528 adolescents, aged 13-16 years old, from 10 European cities, with no inclusion of Portugal cities, showed that lower aerobic fitness was negatively related to the obesity status and cardiometabolic risk of the adolescent population by around 10% [55]. Furthermore, as already mentioned, in adolescents good cardiorespiratory fitness is positively related to body composition and metabolic syndrome in children and adolescents aged 5–19 years old [56]. Furthermore, low aerobic fitness carries negative consequences for the physical function in all age groups, such as increased metabolic and cardiac disease [57,58] and, in this sense, reveals the importance of keeping physically active in adolescence [59,60,61]. Additionally, keeping satisfactory aerobic fitness levels across the years is inversely related with all causes of mortality, such as cardiovascular disease, cancer, chronic lower respiratory tract diseases, accidents and injuries, Alzheimer’s disease, diabetes mellitus, influenza/pneumonia, nephritis, nephrotic syndrome, or nephrosis [62].

## 5. Study Strengths, Limitations, and Future Perspectives

We consider the following as the strengths of this study. We may highlight the importance of this investigation, which searched to identify how abdominal adiposity and physical fitness can influence the obesity status through the BMIs a specific population of Portuguese adolescents. Furthermore, this study stresses the importance of public policies to promote physical exercise and sports programs to improve physical fitness and to reduce obesity in adolescence, thus avoiding the complications of obesity in later life and improving the quality of life of the population. We highlight some limitations in this study, such as a lack of physical activity/sedentary behavior levels and dietary behavior control, as well as the absence of biochemical variables, which could help to explain the Portuguese adolescent’s BMI in a more elaborate manner. Additionally, not all the measures from FITescola^®^ were considered, such as abdominal resistance, upper limb strength, and lower and upper limb flexibility. For future investigations, we encourage researchers to keep analyzing bigger samples of Portuguese children and adolescents and to use these gaps to improve the analysis and contribute to increasing this investigation.

## 6. Conclusions

We conclude that higher age and abdominal adiposity were positively related to the higher BMIs of adolescents. Furthermore, lower horizontal jump distances and worse times on the 40 m maximal velocity were inversely related with a higher adolescent BMI, and lower aerobic fitness was inversely related with a higher adolescent BMI.

## Figures and Tables

**Table 1:** Characteristics of the sample.

Characteristics of the Sample (*N* = 654)	$\mathrm{Age}\;(\bar{X}\;\pm \;\mathrm{SD})$	$\mathrm{Height}\;(\bar{X}\;\pm \;\mathrm{SD})$	$\mathrm{Body}\;\mathrm{Weight}\;(\bar{X}\;\pm \;\mathrm{SD})$
Male, *N* = 334 (51%)	14.6 ± 2.2 y/o	167.4 ± 79.9 cm	58.4 ± 15.5 kg
Female, *N* = 320 (49%)	13.01 ± 2.06 y/o	158.3 ± 11.6 cm	54.38 ± 15.7 kg

Note: Sample characteristics are described in percentage values (%), mean ($\bar{X}$), and standard deviation (SD); y/o, years old; cm, centimeters; kg, kilograms.

**Table 2:** Characteristics of the study by obesity category (*n* = 654).

Variables	*N* (654)	Normal Weight	Overweight	Obesity	*X* ^2^	*p*-Value
*Gender*						
Male	*N* = 334 (51%)	242 (71%)	55 (18%) *	37 (11%)	34.66	*p* < 0.001
Female	*N* = 320 (49%)	219 (68%)	65 (20%) *	36 (12%)	36.67	*p* < 0.001
BMI by Age (15.7 ± 1.21)						
10 y/o	64 (10%)	45 (70%)	8 (13%)	11 (17%) *	46.81 37.05	^Ow^11, 12, 13, 15, and 18 y/o > 10, 14, 16, 17 y/o *p* < 0.001 ^Obes^10, 11, 13, and 14 y/o > 12,15, 16, 17, and 18 y/o *p* < 0.001
11 y/o	81 (12%)	50 (62%)	20 (25%) *	11 (14%) *		
12 y/o	53 (9%)	37 (70%)	11 (21%) *	5 (9%)		
13 y/o	85 (13%)	51 (60%)	22 (26%) *	12 (14%) *		
14 y/o	77 (12%)	57 (74%)	11 (14%)	9 (12%) *		
15 y/o	117 (18%)	83 (71%)	24 (21%) *	10 (9%)		
16 y/o	94 (14%)	77 (82%)	11 (11%)	7 (7%)		
17 y/o	60 (9%)	47 (80%)	8 (12%)	5 (8%)		
18+ y/o	23 (3%)	15 (68%)	6 (23%)	2 (9%)		
Abd. Circumf. by Age		No Risk	Risk			
Male	*N* = 334 (51%)	284 (84%)	45 (16%)		88.311	*p* < 0.001
Female	*N* = 320 (49%)	151(47%)	164 (53%) *			
10 y/o	62 (10%)	45 (7%)	19 (3%)		86.406	11 y/o, 15 y/o, *p* < 0.001
11 y/o	82 (13%)	39 (6%)	43 (7%) *			
12 y/o	54 (8%)	24 (4%)	30 (5%)			
13 y/o	84 (13%)	61 (9%)	23 (4%)			
14 y/o	77 (12%)	67 (10%)	10 (2%)			
15 y/o	116 (18%)	52 (8%)	64 (10%) *			
16 y/o	94 (14%)	79 (11%)	15 (3%)			
17 y/o	58 (9%)	50 (8%)	8 (1%)			
18+ y/o	22 (3%)	18 (2%)	6 (1%)			

Note: *X*^2^, chi-square statistics; y/o, years old; ow, adolescents from both gender with overweight; obes, adolescents from both gender with obesity; *statistical significance at* * *p* < 0.001.

**Table 3:** Hierarchical multivariate linear regression analyses of predictor variables of adolescents’ BMIs adjusted for age and both genders.

Predictor Variables (*n* = 654)	Model 1	Model 2
Age (years)	0.255 **	0.268 **
Gender		−0.207
Abdominal adiposity (cm)	0.313 **	0.313 **
Horizontal jump (cm)	−0.100 *	−0.900 *
40 m velocity (Sec)	0.274 *	0.264
Aerobic fitness (turns)	−0.250 **	−0.250 **
R^2^	0.75	0.75
Adjusted R^2^	0.74.6	0.74.6
F	382.2 **	320.2 **
R^2^ variance*^RSS^*	3202.8	3196.7

Note: Hierarchical multiple linear regression of abdominal obesity, physical fitness, and adolescents’ BMIs. Model 1, adjusted for age; model 2, adjusted for age and gender, *statistical significance at* * *p* < 0.05; ** *p* < 0.001; *RSS*, residual sum of squares variances for models 1 and 2.

## Data Availability

Data are available under request to the contact author.
